# Fast, easy and green method for the first quantification of niacinamide in serums and creams by digital image analysis using iron (III) chloride

**DOI:** 10.1007/s00604-026-08121-4

**Published:** 2026-05-22

**Authors:** Ainhoa Lambarri, Miren Ostra, Ane Bordagaray, Rosa Garcia-Arrona, Maider Vidal

**Affiliations:** Department of Applied Chemistry, University of the Basque Country (EHU), 20018 Donostia/San Sebastian, Spain

**Keywords:** Niacinamide quantification, Complex formation, HPLC, Digital image analysis, Solid-phase extraction, Colorimetry

## Abstract

A green digital image photometry method for the quantification of niacinamide in commercial cosmetic serums and creams is described. The method is based on the formation of an orange complex between niacinamide and iron (III) chloride in microplates under LED backlighting. The analytical signal was defined as (255 − B), where B corresponds to the blue channel intensity in the RGB colour model. Calibration was performed using microplate-based standards, and the method was applied to commercial cosmetic samples. Relative errors compared to HPLC-DAD were lower than 10.0%, and an elliptical joint confidence region test indicated no significant differences between slopes and intercepts and the ideal values of 1 and 0 at the 95% confidence level. Paired Student’s t-test and F-test confirmed no significant differences in agreement and precision between methods. Deviations from labelled concentrations were generally below 6.0%, with a maximum value of 14.0%. Precision, evaluated over two months, showed relative standard deviations below 9.0%. Recovery values ranged from 84 to 114%. UV–Vis spectrophotometry was used to support the validation of the colorimetric response of the niacinamide–FeCl₃ system. The proposed approach was applied to rapid screening of niacinamide in cosmetic products and compared with a reference HPLC-DAD method.

**Graphical Abstract:**

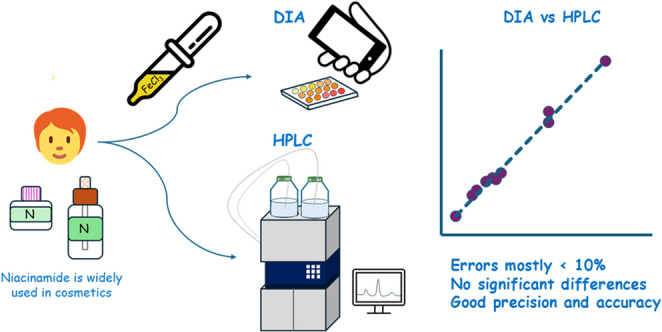

**Supplementary Information:**

The online version contains supplementary material available at 10.1007/s00604-026-08121-4.

## Introduction

Niacinamide, also known as nicotinamide or the amide of nicotinic acid, is a derivative of vitamin B3 that the body requires for proper functioning and the maintenance of healthy cells. This compound is used in the treatment of diabetes and various skin problems, such as redness, acne and dehydration. It is also being investigated for its potential in the treatment of certain types of cancer [1].

Nowadays, millions of people use cosmetics such as makeup, creams and serums daily. Numerous articles have confirmed that niacinamide is commonly included in beauty products due to its ability to reduce blemishes and enhance skin hydration [2, 3]. For instance, a 2002 study demonstrated that a cream containing niacinamide produced better results than a similar cosmetic formulation without it [4]. Additionally, a 2013 study suggested that lotions containing niacinamide may offer benefits in repairing and protecting the skin against UV-induced damage [5].

Due to the widespread use of cosmetics by the general population, their analysis has become one of the most important areas of research in recent years. This can be performed using different technics and analytical instruments, with mass spectrometry - typically coupled with chromatographic techniques such as gas chromatography (GC) or high-performance liquid chromatography (HPLC) - being the most widely used for detecting ingredients [6], ensuring quality control, tracing potential risks [7], verifying authenticity [8] and evaluating efficacy [9–11]. HPLC-DAD has also been employed for the identification and quantification of multiple analytes, including niacinamide, in various samples at different wavelengths [12], such as in multivitamin preparations [13] or urine analysis [14]. In many cases, the sample cannot be analysed directly and requires prior treatment. Common sample preparation methods include liquid-liquid extraction (LLE) [15], solid-phase extraction (SPE) and related approaches [16]. Other techniques such as colorimetric analysis [17] and ion-selective sensors [18] have been also employed in cosmetic analysis.

UV-visible spectrophotometry is the most used equipment for colorimetric analysis. It enables the quantitative analysis of chemical species that absorb electromagnetic radiation in the ultraviolet and visible regions. This technique requires the use of a photometer or spectrophotometer, which consists of a radiation source, a monochromator, a photodetector and an amplifier. In this research, a double-beam spectrophotometer has been used, allowing simultaneous measurement of both the blank and the sample. However, many determinations that have traditionally been performed using UV/Vis spectrophotometry are now increasingly carried out though digital image analysis.

Digital image analysis (DIA) has become a crucial tool for the quantification of chemical compounds. Its application ranges from pharmaceutical analysis using smartphones [19] to various industrial and biomedical fields. Among the advantages of DIA, it is the minimal use of reagents, which aligns with the principles of green chemistry [20]. In this context, DIA can be implemented in a photometric mode (digital image photometry), where the analytical signal is based on colour intensity measurements extracted from digital images. To ensure accurate visualization and analysis, it is important to enhance image quality by removing noise, correcting distortion, increasing contrast and sharpening relevant features. The most common model used in DIA is RGB colour space (red, green, blue) [21], although other models such as CMYK (cyan, magenta, yellow, black) are also employed in certain contexts.

In colorimetric digital image analysis, it is essential to select a colorimetric reaction that offers sufficient sensitivity and selectivity for the target analyte – often one of the main challenges in method development. While many studies report the use DIA without prior purification of the samples, the presence of potential interferences in complex matrices may necessitate the implementation of extraction or separation techniques prior to image acquisition. In this context, microextraction strategies have gained increasing attention due to their simplicity, low solvent consumption, and compatibility with miniaturized analytical approaches. Techniques such as solid-phase microextraction (SPME), dispersive liquid–liquid microextraction (DLLME), and related methods have been widely applied in environmental, food, and pharmaceutical analysis. For instance, solid-liquid phase microextraction (SLPME) has been coupled with DIA for the quantification of dithiocarbamates in food samples [22].

In this work, a colorimetric approach combined with digital image photometry is proposed for the quantification of niacinamide in cosmetic serum and cream samples. The method is based on a simple chemical reaction between niacinamide and FeCl₃, which produces a measurable colour change associated with analyte concentration and can be quantitatively evaluated through RGB image analysis. In addition, the applicability of the method to different cosmetic matrices is considered, considering that minimal sample preparation may be required depending on the sample composition. This approach aims to provide a simple, low-cost, and accessible alternative to conventional analytical techniques for routine analysis.

The aim of this work is to develop and validate a simple, low-cost, and environmentally friendly method for the quantification of niacinamide in cosmetic serum and cream samples based on digital image photometry. The analytical performance of the proposed method is evaluated and compared with a reference HPLC-DAD method to assess its accuracy, precision, and applicability to real samples. The proposed method is intended as a complementary tool for rapid screening rather than a replacement for reference methods.

## Experimental

### Materials

Niacinamide (ITW Reagent, 100%*)* was used to prepare standard solutions for HPLC, spectrophotometry and image analysis. FeCL_3_ (Fluka Chemika, 98%) was used as the colorimetric reagent for both spectrophotometry and digital image analysis. A mixture of methanol (Labkem, 99,9%) and mili-Q water were used as the HPLC mobile phase.

A total of nine serum samples (*Skn*, *Oly*, *Rvu*, *Ctr*,* Ord*, *Bet*, *Rvl*, *Ord R*, and *Ins*) and three cream samples (*Num*, *Msn*, and *Stm*) were analysed. The declared niacinamide content was indicated on the labels of all samples except for three serums (*Skn*, *Oly*, and *Rvu*), which stated the presence of niacinamide but did not specify its concentration. Two serums (*Ord R* and *Ins*) reported no niacinamide content and were considered as free-niacinamide samples. The labelled niacinamide concentrations were 5% for *Ctr*, 10% for *Ord* and *Bet*, and 15% for *Rvl* in serums, and 3%, 4%, and 5% for *Num*, *Msn*, and *Stm*, respectively, in creams.

### Standards and samples

A 0.50% (w/v) stock solution of niacinamide was prepared in water. For the reference method, HPLC-DAD, calibration was performed using seven standards ranging from 0.0025% to 0.040% (w/v), prepared in volumetric flasks. Sample solutions were appropriately diluted, filtered through a 0.45 μm nylon membrane filter, and injected into the system.

For the proposed digital image photometry method, calibration standards were prepared in a 96-well microplate, covering a concentration range from 0.0400% to 0.125% (w/v), including also a blank. A 1.6% (w/v) stock solution of FeCl_3_ was used as the colorimetric reagent, and 80 µL were added to each well for both standards and samples. The final volume in each well was adjusted to 400 µL with water by adding the appropriate amount of solvent to achieve the desired niacinamide concentration. The analytical signal was obtained from digital images as colour intensity based on the blue channel of the RGB system, defined as (255 − B).

In the case of the UV-Vis spectrophotometry, calibration was carried out in 25.00 mL volumetrics flasks using eight standards, starting at 0.010% (w/v) niacinamide and increasing in 0.010% (w/v) increments. To each flask, 2.00 mL of the FeCl_3_ stock solution were added and they were finally diluted to volume with water.

For the serum samples, 250 mg of each product were dissolved in 25.00 mL of water under agitation in a beaker using a glass rod (stirring) to obtain 1:100 (w/v) solutions. For HPLC analysis, 3.00 mL of these solutions were further diluted to 25.00 mL, filtered (0.45 μm), and analysed. For DIA, 320 µL of the 1:100 solutions were directly added per microplate well. In the case of the *Ctr* serum, due to its low niacinamide content, a more concentrated solution (1:62.5) was prepared dissolving 400 µL in 25.00 mL. For cream samples, 200 mg of each product were dissolved in 10.00 mL of water under agitation in a beaker using a glass rod (stirring) to obtain 1:50 (w/v) solutions. For HPLC analysis, 2.50 mL were diluted to 10.00 mL and filtered prior injection. For DIA, the same 1:50 solutions were filtered to remove insoluble components.

Solid-phase extraction (SPE) was performed to selected serum samples (*Oly*, *Ctr*, and *Ord*) to evaluate matrix effects. SPE cartridges (HyperSEP^™^ C18) were conditioned with 9.00 mL of methanol. Then, 3.00 mL of the 1:100 sample solutions were loaded onto the cartridges. Elution was performed sequentially using 3.00 mL of milli-Q water, 9.00 mL of a 50:50 MeOH: H_2_O mixture, and finally 6.00 mL of pure MeOH. The combined eluate (21.0 mL) was diluted to 25.00 mL with water (named *SPE solution*) prior to analysis. For the *Ctr and Ord* samples, 5.00 mL of the *SPE solution* were diluted to 25.00 mL, whereas for the *Oly* sample, 3.00 mL were diluted to 10.00 mL. All analyses were performed in triplicate.

Recovery analyses were performed for *Ctr*, *Ord*, and *Bet* serum samples at two concentration levels using the digital image photometry method. Three aliquots of each sample were prepared at an initial concentration of 0.080% (w/v). Known amounts of niacinamide standard solution were added to two aliquots to obtain final concentrations of 0.100% and 0.120% (w/v). Image acquisition was then carried out and recovery values were calculated accordingly.

### HPLC-DAD

HPLC-DAD was used as the reference method. Niacinamide quantification was performed using a SHIMADZU LC-20AD-HPLC equipped with a diode array detector (SPD-M20A). Separation was carried out on a SunFire C18 column (5 μm particle size). For the chromatographic analysis a injection volume of 20 µL and a flow rate of 1.00 mL/min were used. The mobile phase was made by mixing Mili-Q H_2_O (A) and Methanol (B) with the following gradient: at 0.00 min 90% B, at 4.50 min 90% B and at 8.50 min 10% B. Note that these settings may require adjustment depending on the specific instrumentation employed. Under the reported conditions niacinamide, with a maximum absorbance at 261 nm, eluted at a retention time of 5.9 min, with a total run time of 10,5 min.

### Spectrophotometry

A PG instrument T92 + double-beam UV-vis spectrophotometer was used for the spectrophotometric analysis. This technique was used to estimate the minimum concentration of niacinamide above which iron (III) chloride can be reliably used as a colorimetric reagent, and to assess whether the colour change as a function of niacinamide concentration follows a linear trend. The pH of the formed coloured complex was measured using a Crison Basic 20 pH meter (Crison, Spain), which had been calibrated with pH 4.00, 7.00 and 9.00 standard solutions.

In addition, the continuous variation method was performed to confirm the formation of a complex between niacinamide and FeCl_3_ [23]. Among the different methods available to determine the stoichiometry of a complex, this one is considered the most reliable because it maintains the total concentration constant, minimizes the effect of competing equilibria, and allows the predominant species to be clearly identified even when multiple complexes coexist in solution. The same double-beam spectrophotometer was used for this analysis. Solutions of niacinamide and FeCl_3_ (0.1 M each) were prepared. Nine mixtures were then prepared in laboratory vials by combining the following volume ratios (mL niacinamide : mL FeCl_3_): 1:9, 2:8, 3:7, 4:6, 5:5, 6:4, 7:3, 8:2 and 9:1. Subsequently, the mixtures were allowed to stand for 1 h and 30 min, after which absorbance values from 400 nm to 700 nm were recorded by transferring 0.40 mL of each solution to a cuvette and adding 2.1 mL of milli-Q water.

### Digital image analysis

For the image analysis conducted in this study, a LED light plate was used to illuminate the microplates uniformly during photographies, thereby enhancing colour consistency across the images. In this case a white-light LED plate from the TSOCOS brand was used. To minimize interferences from ambient light, the microplate was enclosed within a plain cardboard box. (42.0 × 31.5 × 21.2 cm). Image analysis was performed using Color Detector^®^ mobile application, which allows the quantification of red, green and blue (RGB) components of individual pixels. A detection radius of 10, which is equivalent to 314 pixels per analysed well, was selected. Although this specific application is now longer supported, several alternative tools are available for free, such as RGB Color Detector^®^, Color Name AR^®^, and Color Picker: RGB Detector^®^. Since the method is based on standard RGB channel extraction, it is not dependent on a specific software platform in terms of relative analytical response, provided that a consistent image acquisition and processing workflow is used for all measurements.

In this work, digital image analysis was applied in a photometric mode (digital image photometry), where the analytical signal is based on colour intensity. Specifically, the blue channel (B) of the RGB colour space was selected, and the analytical response was defined as (255 − B), which is proportional to the analyte concentration.

**Fig. 1:**
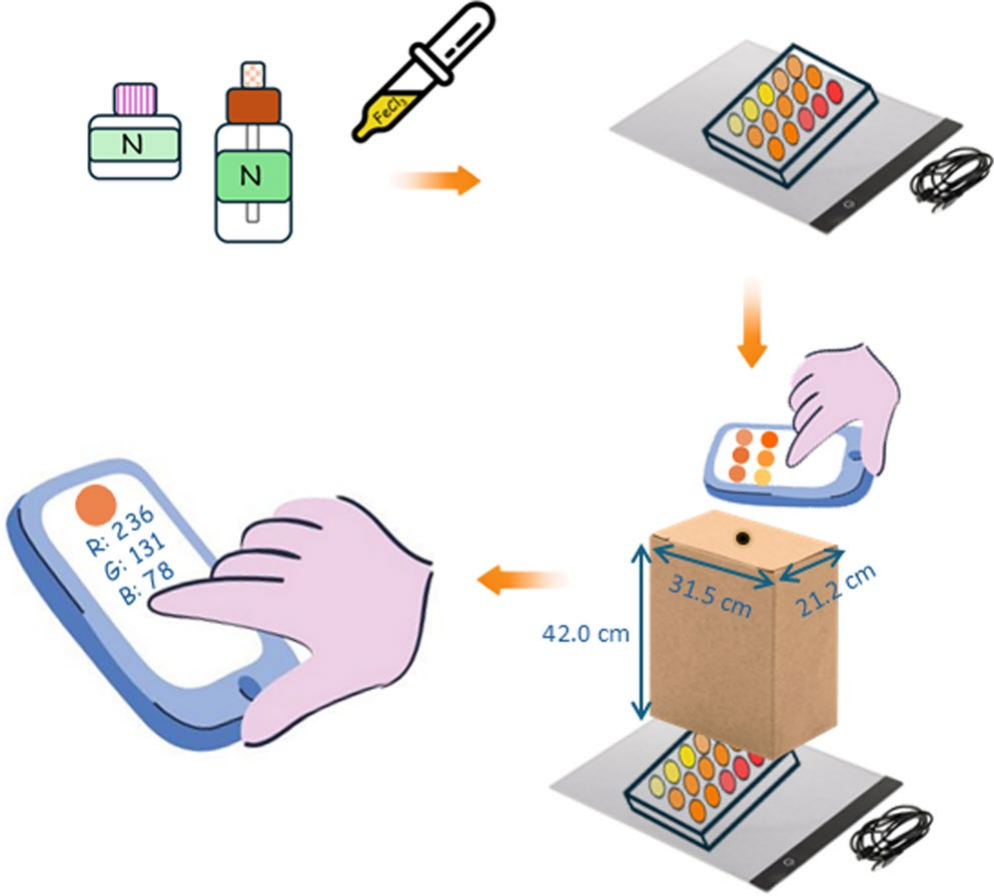
Experimental setup used to capture images after colour development from the reaction between niacinamide-containing samples and the chromogenic reagent in a microplate. The microplate is placed on a white LED light panel and covered with a cardboard box to minimize ambient light interference A smartphone camera is positioned above the box opening to capture images of the microplate. Finally, RGB colour values for each well are extracted using a mobile application (RGB Detector^®^)

 All images were captured using an iPhone 12 pro max, and the camera settings during the acquisition were as follows: aperture f/16, automatic ISO, autofocus mode, 12 MP resolution and a focal length of 26 mm. Fig. 1 presents a schematic of the photography setup and the equipment used.

Experimental setup used for image acquisition after colour development from the reaction between niacinamide-containing samples and the chromogenic reagent in a microplate. The microplate is placed on a white LED light panel and covered with a cardboard box to minimize ambient light interference. A smartphone camera is positioned above the box opening to capture images of the microplate. Finally, RGB colour values for each well are extracted using a mobile application (RGB Detector^®^).

## Results and discussion

Since all the work presented below is based on the reaction between niacinamide and FeCl₃ to form an orange complex, one of the first aspects to rule out is the possible interference of other compounds, such as iron hydroxides - which could have a similar colour- that might form in the medium. For this reason, pH control during the reaction is important. The pH of a 1.6% (w/v) FeCl₃ solution was 1.74, while the pH of a 0.1% (w/v) niacinamide solution was 6.27. After mixing both solutions and allowing sufficient time for the stable formation of the complex (1 h and 30 min), the measured pH was 3.05. Since potential iron hydroxide complexes form at basic pH values, their formation can be excluded under the experimental conditions used in this work.

**Fig. 2:**
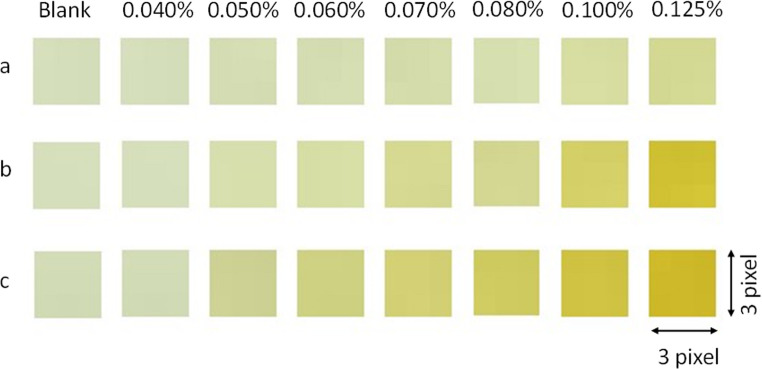
Photographs of the cropped wells containing a blank (column 1) and calibration standards from 0.04% (column 2) to 0.12% (column 8). (**a**) at the time of preparation, (**b**) after 1 h and 30 min and (**c**) after 3 h from preparation

The initial procedure using DIA involved optimizing the reaction time between niacinamide and FeCl₃ to ensure adequate colour development and sufficient analytical sensitivity within a reasonable timeframe. Once the calibration standards for DIA were prepared in the microplate - in triplicate to minimize errors – this was placed on a LED light plate and photographed every 15 min over a period of 3 hours. Figure 2 illustrates the progression of the colour change in some of the calibration standards over time. For this purpose, a small region of 3 × 3-pixel area was extracted from each well. This extraction was made with the software MatLab^®^.

The RGB values for each cropped image of each plate were calculated. Using the *Color Detector*^®^ application, a detection radius of 10 (314 pixels) within each plate was selected to determine the red, green and blue intensity levels. Several RGB channels combinations were considered for regression. As reference, the approaches suggested by Soares, S. et al. [24] were considered, and the best linear result was reached when 255 – B was selected as analytical signal, *where B represents* the blue values of the selected pixel for each standard (or sample subsequently).

1 $$\:S\:\left(Analytical\:signal\right)=255-B$$

When plotting the slopes of the different calibrations as a function of analysis time (at 15-minute intervals), it was observed that the slopes consistently increased, although the incremental gain became negligible at longer times. The criterion for selecting the optimal analysis time was based on the point at which this increase accounted for less than one third of the total gain. According to this criterion, the signal effectively stabilized at 90 min, beyond which further variations were minimal and did not significantly affect the final result. Notably, this analysis time also corresponded to the calibration with the highest R² value (0.9942). Although this duration may appear considerable, the possibility of measuring all standards and samples within the same microplate notably enhances the overall efficiency of the analysis.

The final obtained calibration at the optimized time of 90 min is showed in Supplementary Fig. 1 of the supplementary material. A slope of 1865 ± 43 (1/% w/v) and an intercept of −19 ± 4 (dimensionless) were obtained. This calibration shows the mean values and standard deviations from standards measured from different images measured at different days, which shows overall method reproducibility. The blank measurement was excluded from the calibration because its signal cannot be considered a simple additive contribution. The blank (yellow) and the standards (orange) exhibit different chromatic properties, leading to a non-equivalent response in the blue channel when using (255 − B) as the analytical signal. Consequently, its inclusion worsened the linearity and did not provide any additional analytical benefit.

In any case, although measurement time is not considered critical provided that sufficient colour development and adequate sensitivity are achieved, and that standards and the samples are captured together in the same image under identical conditions, a reaction time of 90 min was selected as the optimized and fixed condition for all subsequent measurements.

The limit of detection (LOD) and limit of quantification (LOQ) for this analysis were calculated using equations LOD = 3.3*S_*r*_ */m* and LOQ = 10*S_*r*_ */m*, where *s*_*r*_ represents the standard error of the regression and *m* the slope. The resulting values were 0.0072% (w/v) for the LOD and 0.022% (w/v) for the LOQ.

**Fig. 3:**
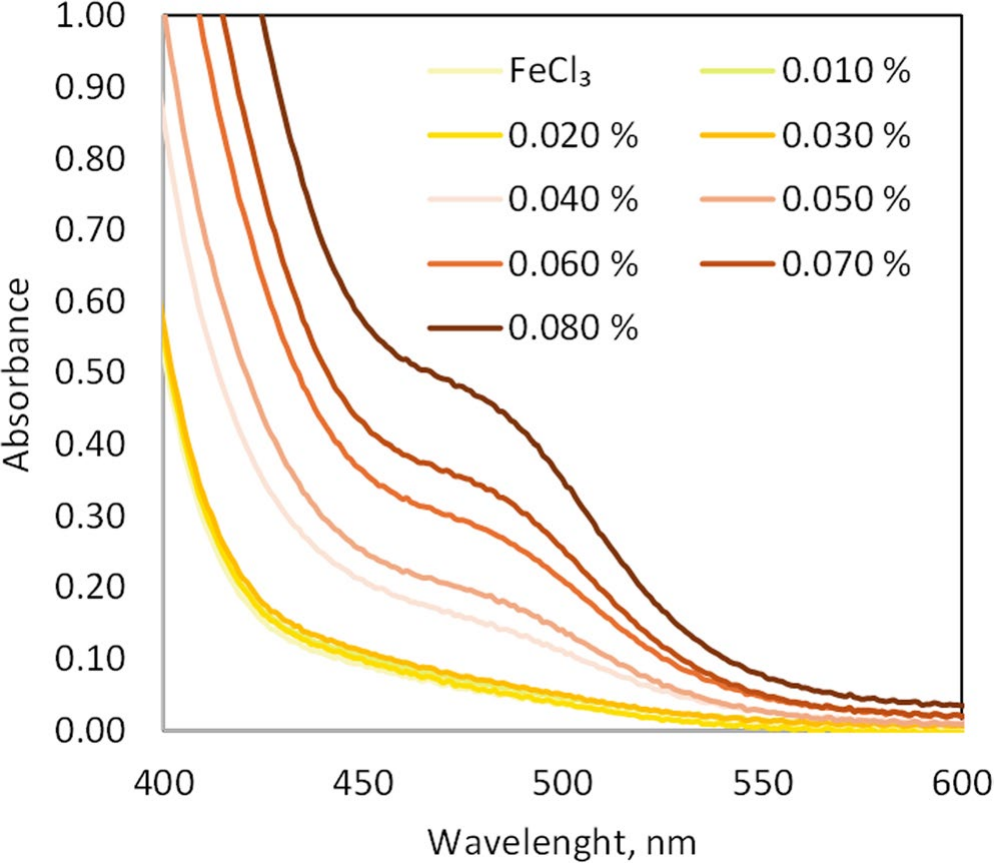
UV–Vis spectra of Niacinamide–FeCl₃ complexes at increasing niacinamide concentrations (0.010–0.080%, w/v). FeCl_3_ concentration keeps constant at 0.13*% (w/v)*

UV–Vis spectrophotometry was employed to support and validate the results obtained by digital image photometry (DIA), confirming the trend observed in the colorimetric response of the niacinamide–FeCl₃ complex. Spectra of the complexes formed were also recorded by UV/Vis spectroscopy at various niacinamide concentrations, ranging from 0.010% to 0.080%. As clearly shown in Fig. 3, noticeable spectral changes compared to FeCl₃ - which displays a yellow colour in solution - are only observed from concentrations of 0.030% onwards. This finding further supports the LOQ value obtained by DIA for niacinamide determination.

Once the standards were analysed by UV-Vis spectrophotometry for preliminary validation of the colorimetric response, both standards and samples were subsequently examined together with the samples using HPLC-DAD as the reference method for final method validation.

**Fig. 4:**
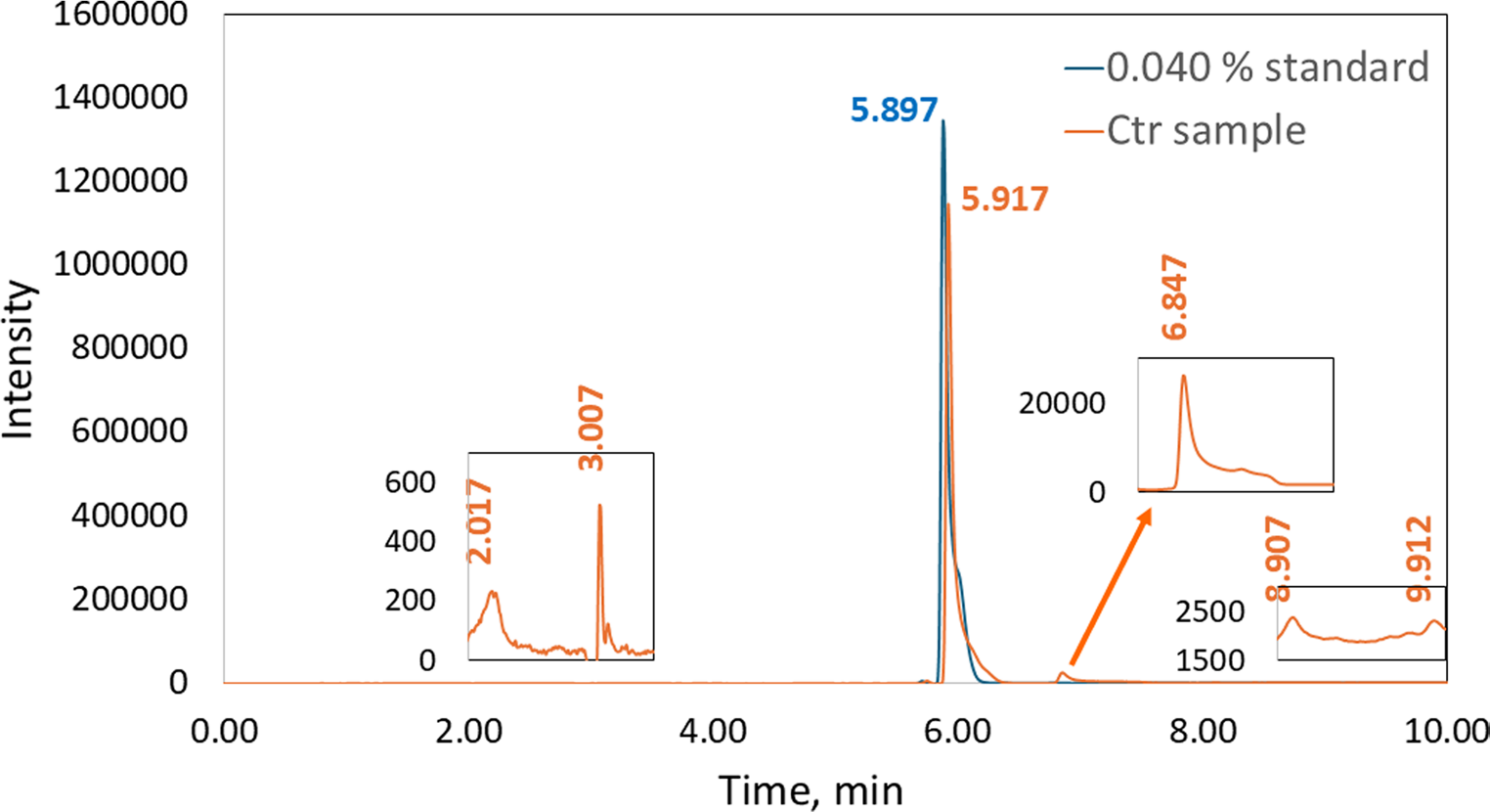
Comparative analysis of the 0.040% standard and the chromatogram of the *Ctr* sample. As can be observed, the *Ctr* sample shows several additional peaks besides the niacinamide peak at approximately 5.9 min, which correspond to other compounds present in the sample. At the wavelength used in the HPLC analysis, the intensity of these peaks is low

As shown in Fig. 4, the niacinamide standards produced a single peak at approximately 5.9 min. In contrast, several small peaks were observed in the chromatogram of the *Ctr* sample in addition to the niacinamide peak. However, these peaks were almost negligible and were barely distinguishable from the baseline at the selected wavelength, 261 nm.

For the HPLC analysis, the LOD and LOQ were calculated using the same approach as in the DIA analysis. This is shown in Table 1. The accuracy and precision of both DIA and HPLC methods were also evaluated. Accuracy was assessed in triplicate using two standard concentrations: 0.060% and 0.100% for DIA, and 0.010% and 0.030% for HPLC. Accuracy and precision were evaluated in a similar manner by analysing the same standards in triplicate on different days (inter-day precision) for HPLC and on same image (intra-day precision) and on different images (inter-image precision) for DIA. The different images in DIA were captured on different days. The corresponding results are presented in Table 1. As can be seen, intra-image accuracy (RSD ≤ 3.1%) and precision (RSD ≤ 2.9%) in DIA are slightly better than those obtained when different images are compared (RSD between 3.4% and 8.1%). This is why it is recommended to include calibration standards with the samples to measure in the same image. In addition, the results obtained by HPLC are only slightly better than those of DIA inter-image. (RSD ≤ 7.5%).

**Table 1:** Several figures of merit obtained by DIA for the determination of niacinamide, and method validation parameters (accuracy and precision). HPLC results have been added for comparison

	DIA			HPLC	
R^2^	0.9942			0.9983	
LOD (%)	0.0072			0.0036	
LOQ (%)	0.025			0.011	
Linearity (%)	0.040–0.125			0.0075–0.040.0075.040	
	Standard, % (w/v)	Intra-image (%)	Inter-image (%)	Standard, % (w/v)	Inter-day (%)
Accuracy	0.060	3.1	8.1	0.010	0.11
	0.10	2.1	3.4	0.030	5.1
Precision	0.060	2.9	4.7	0.010	6.0
	0.10	2.0	8.8	0.030	7.5

Additionally, the LOD and LOQ values calculated using HPLC are approximately two times lower than those estimated via image analysis. While this difference is expected, it does not represent a limitation for the intended analysis, as the niacinamide concentrations in the samples range between 1% and 15%, according to product labels.

Following method validation, the samples were analysed, and the results are summarized in Table 2 alongside the corresponding HPLC measurements and the label-declared values. Notably, these results were obtained without applying any prior extraction procedure to the samples. It is important to highlight that, to ensure consistency across images, calibration standards were always included on each microplate during sample analysis by DIA.

**Table 2:** Comparison of the results obtained for niacinamide concentrations using DIA and HPLC (expressed as mean ± standard deviation), along with the percentage declared on the product label (serum or cream)

Niacinamide in Serums, % (w/w)			
Sample	**DIA**	**HPLC**	**LABEL**
*Ctr*	4.9 ± 0.1	4.9 ± 0.5	5
*Ord*	10.6 ± 0.3	10.2 ± 0.5	10
*Bet*	9.7 ± 0.6	10.2 ± 0.4	10
*Rvl*	15.0 ± 0.1	15.6 ± 0.6	15
*Skn*	4.8 ± 0.2	5.20 ± 0.02	Unspecified
*Oly*	6.09 ± 0.06	1.56 ± 0.01	Unspecified
*Rvu*	3.81 ± 0.09	3.4 ± 0.1	Unspecified
Niacinamide in creams, % (w/w)			
Sample	DIA	HPLC	LABEL
*Num*	3.4 ± 0.1	3.0 ± 0.1	3
*Msn*	4.57 ± 0.02	4.31 ± 0.06	4
*Stm*	5.3 ± 0.2	5.68 ± 0.08	5

label (serum or cream)

The data show that the niacinamide percentages obtained by DIA were comparable to those obtained by HPLC, with relative errors ranging from 0 to 13% (except for one case, which had an error of 290%). Relative errors with respect to the label-declared values ranged from 0% to 14% (71% between 0 and 6%). The niacinamide content of the *Skn*, *Rvu*, and *Oly* samples was also quantified. While the *Skn* product explicitly declared its niacinamide content on the front label (in addition to 0.3% retinol concentration), neither *Rvu* nor *Oly* indicated its presence on this front packaging. Nevertheless, niacinamide was listed among the primary ingredients in both products. For *Skn*, the relative error compared to the HPLC result was nearly 8%. For *Rvu* similar values of niacinamide (between 3 and 4%) were found by both HPLC and DIA (12% error), whereas *Oly* showed substantial discrepancies between the results obtained by DIA and HPLC (error of 290%). It was hypothesized that in this case other components of the formulations, or their interactions, might be interfering with the FeCl₃ colorimetric reagent. All listed ingredients (> 20) correspond to standard INCI (International Nomenclature of Cosmetic Ingredients) components commonly used in cosmetic serum formulations, with no evidence of brand-specific or proprietary ingredients, making it difficult to pinpoint the source of the discrepancy.

To address the observed discrepancies between DIA and HPLC results, solid-phase extraction (SPE) was performed on *Oly* and additional samples to assess the efficiency of the extraction procedure and minimize potential matrix interferences affecting the colorimetric response of the FeCl₃ complex. The SPE protocol followed the methods described in the *Standards and samples* section. After extraction, the samples were reanalysed by both DIA and HPLC to evaluate the effectiveness of SPE in removing co-extracted species and to confirm the reproducibility and reliability of the analytical signals. Results can be seen in Table 3. *Ctr* and *Ord* gave the same result by HPLC as before SPE, whereas the result for *Oly* changed very little. Respect to DIA, results for *Ctr* and *Ord* showed practically unchanged, but the result for *Oly* varied abruptly more than a 70% (from 6.09 ± 0.06 to 1.58 ± 0.03). This demonstrates the effectiveness of SPE procedure in this sample. In any case, it is not possible to determine which result is closer to the actual niacinamide concentration of *Oly*, since the product label did not specify the exact content. In the absence of a certified reference value for all samples, it is not possible to definitively assess which method provides results closer to the true niacinamide content, as illustrated in the case of the *Oly* sample. Therefore, the necessity of sample pretreatment by solid-phase extraction (SPE) cannot be established a priori. Based on the results obtained, SPE is considered a corrective step to be applied only when significant matrix effects are suspected, as indicated by deviations between DIA and HPLC results or evidence of interference in the colour development reaction. In samples with simpler matrices, SPE was not found to produce significant changes in the determined niacinamide content by either method.

**Table 3:** Niacinamide concentration in serums, in % (w/w), after solid phase extraction (SPE) procedure by HPLC and DIA

Sample	Niacinamide in Serums, % (w/w) after SPE		Label
	DIA	HPLC	
*Ctr*	4.8 ± 0.2	4.9 ± 0.5	5
*Ord*	10.6 ± 0.4	10.2 ± 0.5	10
*Oly*	1.58 ± 0.03	1.4 ± 0.1	Unspecified

Accordingly, SPE is recommended as an optional pretreatment step depending on matrix complexity rather than as a mandatory procedure for all samples.

Furthermore, to evaluate the robustness and reliability of the analytical method, a recovery study was performed on selected samples (*Ctr*, *Bet*, and *Ord*) at two concentration levels and niacinamide concentration was measured by DIA. In addition, a couple of serums containing similar excipients but with no niacinamide declared on the label or list of ingredients, *OrdR* - with retinol as active component- and *Ins* – with hyaluronic acid - were analysed to investigate potential matrix effects and to assess the selectivity of the method. Recovery results for the three samples were in general consistent and within acceptable limits for image-based analysis in the medium concentration range. Specifically, recoveries ranged from 86 to 103% for *Ctr*, 109–114% for *Bet*, and 84–104% for *Ord*. Overall, the results indicate satisfactory method accuracy for all matrices. While *Ctr* and *Ord* showed recoveries close to 100%, *Bet* exhibited a slight positive bias, with recoveries consistently above 109%, suggesting a potential matrix effect or attributable to inherent limitations of image-based quantification. On the other hand, no detectable amounts of niacinamide were found in *OrdR* and *Ins* samples, which demonstrates the robustness of the applied procedure for niacinamide quantitation.

To assess whether there are significant differences between the DIA and HPLC results, a joint confidence ellipse test for the slope and intercept was performed [25]. All results of Table 2, excluding the *Oly* sample, and *Oly* result of Table 3 were included in this test. This statistical approach is commonly used to compare new analytical methods against reference techniques. For instance, it has been applied to compare UV-VIS spectrophotometry with ion chromatography (IC) for the determination of hypophosphite and phosphite levels in electroless nickel baths [26], as well as to compare DIA with IC for the determination of phosphate in eye drops [27]. The confidence interval for the slope and for the intercept were 1.05 ± 0.11 and − 0.4 ± 0.5, respectively. Given these values, the ideal point (slope = 1 and intercept = 0) falls within the specified ranges. Therefore, no statistically significant differences were observed between the DIA and HPLC methods, although a slight variation between individual values may still be noted. The joint confidence ellipse is shown in the Supplementary Fig. 2.

In addition, a paired Student’s t-test and an F-test were applied to assess possible systematic differences between the mean values obtained by both methods and to compare the precision of both methods based on the variance of replicate measurements. The calculated *t* values were lower than the critical value at the 95% confidence level (*t*_*calc*_ = 0.868 < *t*_*crit*_ = 2.26), indicating no statistically significant differences in accuracy. The calculated *F* values were lower than the critical value (*F*_*calc*_ = 1.84 < *F*_*crit*_ = 3.179), confirming that no significant differences in precision were observed.

Overall, all statistical approaches consistently confirmed the equivalence between the proposed method and the reference HPLC-DAD method in terms of accuracy and precision. It should be clearly stated that the proposed method is intended as a complementary tool for rapid screening purposes rather than as a replacement for reference analytical methods.

### Molecular structure of the formed complex

As previously mentioned, the continuous variation method was employed to determine the stoichiometry of the complex. It is generally considered more reliable when multiple complexes may coexist in equilibrium, as it keeps the total concentration constant and clearly reveals the composition of the predominant species. Following the measurement of absorbance for the ten prepared solutions, the results were plotted in the Supplementary Fig. 3 of the supplementary material. At low ligand fractions ([L]/([L]+[M]) = 0–0.2), the absorbance values remain nearly constant. This behaviour confirms the observation in Fig. 3: at such low ligand-to-metal ratios, the amount of complex formed is below the LOD and LOQ, so the measured absorbance remains effectively constant despite small differences in complex concentration.

According to the results, the absorbance at 460 nm reaches a maximum at $\:x=\left[L\right]/\left(\right[L]+[M\left]\right)\approx\:0.70$, indicating an approximate 2:1 ligand (niacinamide)-to-metal (Fe) stoichiometry. Although the results suggest that the main species present in solution is the ML₂ complex, the possible presence of other minor complexes in equilibrium cannot be ruled out.

It is worth noting that this result differs from the findings of Al-Saif, F.A., et al. [28], who, through elemental analyses including molar conductivity, infrared and UV-Vis spectroscopy, X-ray powder diffraction, and electron spin resonance for the characterization of several coordination compounds, reported the formation of a 1:1 complex between niacinamide and FeCl₃·6 H₂O with the molecular formula [Fe(NA)Cl₃(H₂O)₂]. The observed discrepancy may arise from differences in experimental conditions, such as concentrations, solvent, pH, or the presence of competing equilibria, highlighting that multiple species could coexist in solution under the conditions used in the present study.

### Analytical greenness evaluation of the proposed methods

Considering the growing concerns regarding the environmental impact of chemical methods, it is crucial to assess the environmental sustainability of the developed analytical procedures. Considering the substantial improvements achieved from a Green Analytical Chemistry (GAC) perspective, the greenness of the DIA versus HPLC method was systematically evaluated using the AGREE metric [29].

DIA produces minimal waste and uses either non-toxic reagents or only very small amounts of potentially hazardous chemicals. Furthermore, DIA enables the simultaneous analysis of multiple samples and offers greater portability and miniaturization compared to conventional methods. For that, an AGREE score of 0.75 was obtained for the DIA, while a score of 0.47 was acquired for the HPLC method (both AGREE scores are available in the Supplementary Fig. 4). These results demonstrate that the use of DIA for the analysis of niacinamide is a more sustainable strategy than standard liquid chromatography.

### Comparison with other methods for niacinamide determination

Few studies have reported quantitative measurements of niacinamide, particularly in cosmetic products, which highlights the need for simple and reliable analytical methods for these matrices. The compiled data in Table 4 show the analytical performance of various techniques for determining niacinamide in different sample types. Reported linearity values (R²) are generally high, ranging from 0.99 for densitometric TLC in vitamin tablets to 0.999 for colorimetric detection in dosage forms, indicating strong calibration reliability where reported. Some methods, such as the homemade chromatography system for skincare creams, did not explicitly report R² values; however, estimations suggest similarly robust linearity (0.998–0.999).

**Table 4:** Comparison of different procedures and methods for the determination of niacinamide. Units are reported according to sample matrix: mg L⁻¹ for liquid products and % (w/w) for solid/semi-solid formulations

Method	Sample	Linear range*	*R* ^2^	LOD*	Ref
Densitometric TLC	Vitamin tablets	300–1000^1)^	0.99	10^1)^	[30]
UV spectrophotometry	Pharmaceutical dosage form	8–241^)^	0.998	0.70 − 0.79^1)^	[31]
LC-MS-MS	Human Plasma	0.07–2.0^1)^	0.9963	0.0033^1)^	[32]
Homemade chromatography system	Skincare Creams	0.01–0.1^2)^	Not reported. Estimated: 0.998–0.999	Not reported. Estimated: 0.005^2)^	[33]
HPLC-DAD	Cosmetic formulations	10–200.8^1)^	1.000	10	[12]
Colorimetry with 1- chloro-2,4-dinitrobenzene	Dosage Forms	200–6301^)^	0.999	Not reported. Estimated: 200^1)^	[34]
Colorimetry with FeCl_3_	Serums and Creams	0.0400 to 0.125^2)^	0.9942	0.0072^2)^	This work

*^1)^ mg L^−1 2)^ %, w/w

Limits of detection (LOD) vary considerably depending on both the method and the sample matrix, from as low as 0.0033 mg L⁻¹ using LC-MS/MS in human plasma to values around 10 mg L⁻¹ for densitometric TLC. Several methods did not report LODs, although approximate estimates are provided for comparison. Notably, the present work using FeCl₃ colorimetry in serums and creams achieves an LOD of 0.0072%, comparable to other sensitive techniques for cosmetic matrices, and demonstrates good linearity (R² = 0.9942) over the tested concentration range.

Overall, while highly sophisticated methods such as LC-MS/MS offer superior sensitivity, simpler and more accessible techniques, including colorimetric assays and homemade chromatography, can provide reliable quantitative results when appropriate method validation is applied.

## Conclusions

A simple and cost-effective method has been developed for the determination and quantification of niacinamide in serums and creams. Although it requires a waiting time of approximately 1 h and 30 min to achieve optimal colour development, the method allows for the simultaneous analysis of multiple standards and samples, thereby improving overall time efficiency compared to traditional chromatography techniques. To the best of our knowledge, this is the first-time digital image analysis has been applied to correlate the intensity of the yellow/orange complex formed between FeCl_3_ and niacinamide with the concentration of niacinamide in the samples. The proposed method was compared to High Performance Liquid Chromatography (HPLC), which served as the reference technique.

As expected, the reference method (HPLC) provided superior slightly analytical performance, including stronger linear correlation and lower detection limit. Nevertheless, the use of digital image analysis for niacinamide determination represents a noteworthy advancement. Through a straightforward procedure, and using only 400 µL of sample, it was possible to both identify and quantify niacinamide in aqueous solutions, in some cases yielding better average values and precision than those obtained with HPLC.

Considering the small sample volume, the method demonstrated good reproducibility, with a mean relative standard deviation of 6.5% from HPLC and below 14% from label.

Based on the data obtained from both the reference and the newly developed method, it can be concluded that they are complementary and yield comparable results. Therefore, the findings of this study support the suitability and reliability of the proposed method for the determination of niacinamide in cosmetic samples.

## Supplementary Information

Below is the link to the electronic supplementary material.

Supplementary Material 1 (DOCX 250 KB)

## Data Availability

Data will be made available on reasonable request. The raw experimental data are held by the co-author who performed the experiments.

## Declarations

### Use of generative AI and AI-assisted technologies

During the preparation of this work, the authors used AI-assisted technologies to support language editing. All content was reviewed and edited by the authors, who take full responsibility for the accuracy and integrity of the publication.
