# Supplementary material for: Fast, easy and green method for the first quantification of niacinamide in serums and creams by digital image analysis using iron (III) chloride
Source: Mikrochim Acta. 2026 May 22;193(6):403. doi: 10.1007/s00604-026-08121-4 (PMC13194199; doi:10.1007/s00604-026-08121-4)
Supplement: Supplementary file 1 — Supplementary Material 1 (DOCX 250 KB) [file 604_2026_8121_MOESM1_ESM.docx]

**Supplementary information**

**Fast, easy and green method for the first quantification of niacinamide in serums and creams by digital image analysis using iron (III) chloride**Ainhoa Lambarri, Miren Ostra, Ane Bordagaray, Rosa Garcia-Arrona, Maider Vidal*

Department of Applied Chemistry, University of the Basque Country (EHU), 20018, Donostia/San Sebastian, Spain.

* corresponding author. E-mail address: maider.vidal@ehu.eus

**Supplementary Figure 1.**  Obtained calibration for DIA analysis at 90 minutes after the beginning of the colorimetric reaction. The blank (orange point) is showed, but it was excluded from the calibration because results were worse.

**Supplementary Figure 2.**  Joint confidence ellipse test for niacinamide: comparison between DIA and HPLC methods.


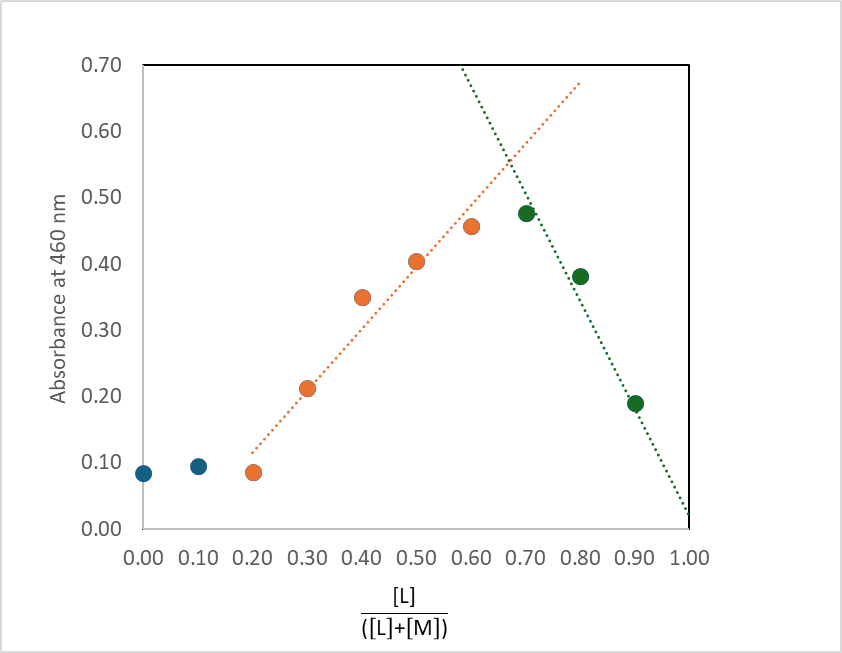


**Supplementary Figure 3.**  Absorbance values of the solutions prepared using the continuous variation method to determine the stoichiometry of the complex. [L] = niacinamide; [M] = FeCl_3_


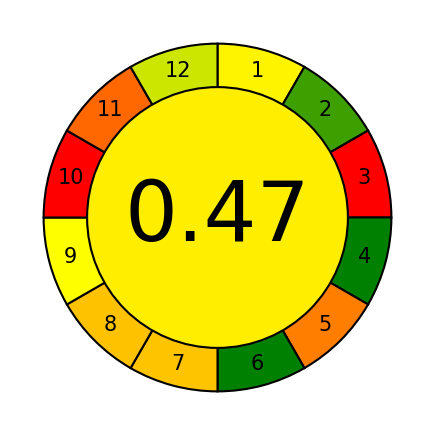

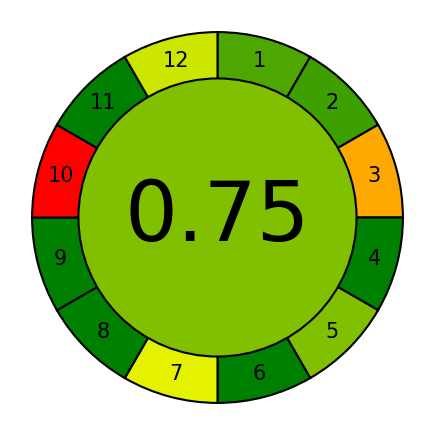


**Supplementary Figure 4.**  Agree Metrics to compare DIA analysis (left) with HPLC (right) for niacinamide detection.
